# Laboratory Plasticware Induces Expression of a Bacterial Virulence Factor

**DOI:** 10.1128/msphere.00311-22

**Published:** 2022-08-22

**Authors:** Philipp Hansen, Kristine von Bargen, Alexandra Jünger-Leif, Albert Haas

**Affiliations:** a Institute for Cell Biology, University of Bonn, Bonn, Germany; University of Kentucky

**Keywords:** *Rhodococcus*, gene expression, microplastic, pathogen, pollution, virulence

## Abstract

Pollution with microplastic has become a prime environmental concern. The various ways in which human-made polymers and microorganisms interact are little understood, and this is particularly true for microplastic and pathogenic microorganisms. Previous reports demonstrated that expression of central virulence-associated protein A (VapA) of the pathogenic bacterium Rhodococcus equi is shut off at 30°C, whereas it is strongly expressed at 37°C, a temperature which may serve as an intrahost cue. Here, we show that cultivation at 30°C in disposable plastic tubes increases mRNA levels of *vapA* 70-fold compared to growth in conventional glass tubes. Strong expression of *vapA* in plastic tubes does not seem to be caused by a compound leaching from plastic but rather by tube surface properties. Expression stimulation during growth in plastic is regulated by the *R. equi* transcription regulators VirR and VirS, indicating that plastic-induced *vapA* expression is (co)regulated through the canonical *vapA* expression pathway. Our observations have important implications for the future analysis and assessment of environmental microplastic contaminations in that they show that, in principle, contact of pathogens with environmental plastic can increase their virulence.

**IMPORTANCE** Millions of tons small plastic pieces (microplastic) find their way into the environment every year. They pose digestive and toxicity problems to various life forms in soil, freshwater, and seawater. Additionally, microplastic offers an opportunity for microorganisms to attach and to become an important part of a “plastisphere community.” The significance of our study lies in the documentation of a sharp increase in production of a central virulence factor by a bacterial pathogen when the bacterium is in touch with certain makes of plastic. Although this feature may not reflect an increased health risk in case of this particular soilborne pathogen, our data disclose a new facet of how microplastics can endanger life.

## INTRODUCTION

Gram-positive Rhodococcus equi (also referred to as Rhodococcus hoagii or Prescottella equi) (1, 2) thrives in many environments but is generally associated with soil. *R. equi* is a facultative intracellular bacterium that can cause severe bronchopneumonia, particularly in young foals and in immunocompromised humans such as AIDS patients (3). Its main target cell is the alveolar macrophage (2), which patrols the lung and ingests inhaled microorganisms. Instead of being killed by macrophages, virulent *R. equi* multiplies within a membrane-bound compartment, the *Rhodococcus-*containing vacuole (4–7). The key to intramacrophage multiplication is the abundant production of virulence-associated protein A (VapA), whose gene is localized to a virulence plasmid. VapA pH-neutralizes the endocytic and phagocytic continua in macrophages, which is a key requirement for intracellular multiplication (6). VapA is produced only at a pathogenetically meaningful, host-associated temperature, i.e., above 34°C, but not at all at environmental temperatures of 30°C or below (8–11), thus preventing the waste of resources during growth in soil.

*R. equi* is routinely cultivated in glass tubes. Here, we present evidence that cultivation in standard labware plastic tubes can strongly increase expression of *vapA*. One possible cause for this effect could be compounds leaching from plastic labware. For example, in a study in 1980, Mithen et al. reported that certain batches of polystyrene petri dishes used in tissue culture are toxic to neurons (12). A landmark paper from Holt’s laboratory (13) described that the leaking of the compounds di(2-hydroxyethyl)methyldodecylammonium and 9-octadecenamide from common laboratory plastic tubes and pipetting tips inhibited the activity of human monoamine oxidase B (hMAO-B) in the test tube. These two compounds are so-called slip agents that are often incorporated into plastic during the manufacture extrusion process. In the following years, additional well-documented cases of plastic-organism interaction were published (14). Considerable public interest in plastic “extractables and leachables” (15) arose when it was shown that the plastic-softening phthalates can reduce male fecundity (16).

Many of the biological effects of plastics are likely mediated by microplastics (<5 mm). They are currently being given wide attention, with billions of potentially biologically active microplastic pieces produced from an estimated 4 to 12 million tons of plastic entering the oceans every year (17). This plastic debris has detrimental effects on sea life such as turtles, fish larvae, marine mammals, mussels (17), and coral reefs, where the likelihood of coral disease is greatly increased in the presence of microplastic (18, 19). Plastic, in particular microplastic, provides a surface for the formation of biofilms. Escherichia coli pathotypes and strains of the human pathogen Vibrio cholerae and of the fish pathogen Vibrio salmonicida have been identified on marine plastic debris (20–23), although it has not been investigated whether the bacteria in these particular samples were virulent. It has been speculated that horizontal gene transfer between pathogenic bacteria could occur within plastic-associated biofilms (18, 24), resulting in accelerated evolution of pathogenic traits (25). However, this hypothesis remains to be tested, and further investigation into microbial gene regulation by human-made plastics is needed, in particular as microplastic also exists in animal intestines, where it could influence transcription of virulence and multiple types of metabolic genes.

Here, we show that the tight temperature regulation of VapA production in broth culture is countermanded. Although this change in expression may not enhance the threat of this particular pathogen to humans or animals, it sets a precedent for a phenomenon which could be a serious microplastic-associated health problem for wildlife, particularly for aquatic animals.

## RESULTS AND DISCUSSION

### Growth of virulent *R. equi* in plastic tubes can induce *vapA* expression at 30°C.

**FIG 1:**
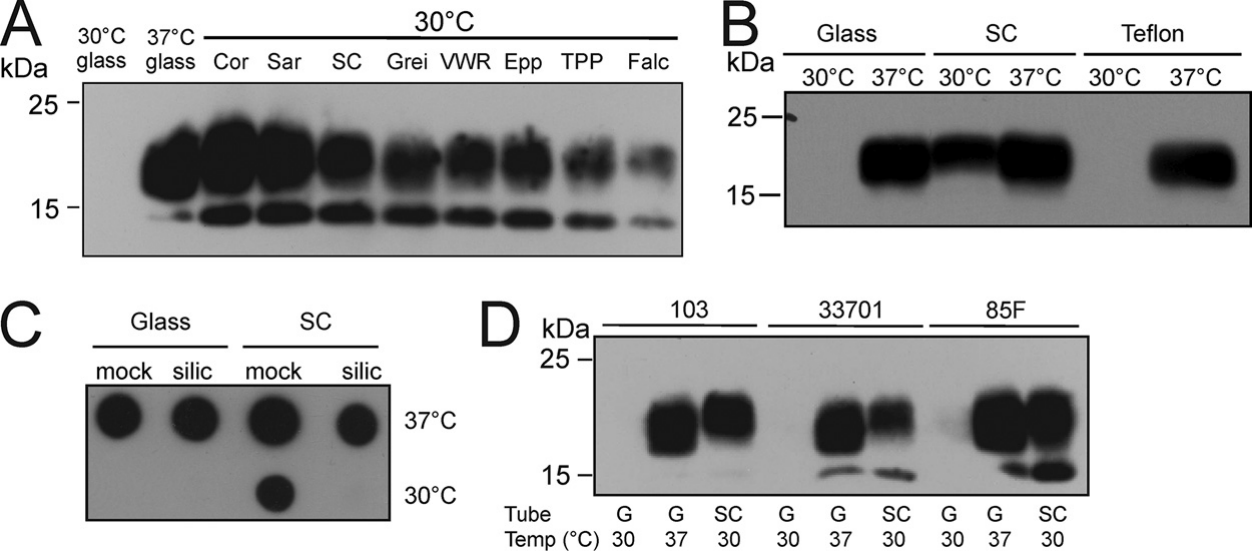
VapA expression in various plastic tubes at 30°C by different *R. equi* isolates. (A) An overnight culture of *R. equi* 33701 at 30°C was diluted into fresh BHI at an OD_600_ of 0.1 and further grown at 30°C or 37°C in glass or plastic tubes for 18 h, as indicated. Proteins from equal numbers of bacteria were analyzed by anti-VapA Western blotting. VapA typically runs as up to three separate proteins with molecular weights between 15 and 18 kDa (11, 49). (B and C) Strain ATCC 33701 was grown in Teflon tubes (B) or in untreated (mock) or siliconized (silic) Sarstedt 13-mL Snap Cap polypropylene tubes (C) at 30°C or 37°C and processed as described above. (D) Cultures of *R. equi* strains 103, ATCC 33701, and 85F were grown in glass (G) or Sarstedt 13-mL Snap Cap polypropylene tubes and processed as described above. The immunoblots are representative of three independent experiments each. Corn, Corning Inc.; Epp, Eppendorf AG; Falc, Falcon Corning Inc.; Grei, Greiner; PP, polypropylene SC; Sar, Sarstedt AG; SC, Sarstedt Snap Cap; TPP, Techno Plastic Products AG; VWR, VWR International.

During our studies on *R. equi* virulence, we observed that VapA was suddenly strongly produced not only at 37°C but also at 30°C, which was in contrast to our earlier observations and those of others (9–11, 26). This new phenomenon apparently coincided with a switch from reusable glass tubes to disposable polypropylene (PP) tubes for the incubation of broth cultures. Follow-up experiments confirmed that VapA was clearly not produced at 30°C in glass tubes but surprisingly was robustly produced in disposable plastic tubes (Fig. 1A). Further analysis of plastic tubes from several vendors and made from different polymers showed that most polypropylene tubes tested induced *vapA* expression at 30°C to some degree, with Falcon tubes being slightly effective and Sarstedt Snap Cap tubes very effective (Fig. 1A, lane SC). Induction was also observed with Techno Plastic Products (TPP) polystyrene and Corning polyethylene terephthalate tubes (Fig. 1A), in spite of their different polymer chemistries, indicating that a general rather than specific plastic property may have been responsible for the effect. However, not all synthetic nonwettable surfaces induce *vapA* expression at 30°C: when tetrafluoroethylene (Teflon) tubes were used for cultivation, VapA production was not detected at 30°C (Fig. 1B). Similarly, siliconized polypropylene tubes did not enhance *vapA* expression (Fig. 1C). For brevity, the observed induction of *vapA* expression at 30°C is referred to as “*vapA* induction” here.

It is important to note that not all tubes, even those from them same batch and from the same producer, showed the same results. For example, different packing units of 13-mL PP tubes from Sarstedt (catalog no. 62.515.028) yielded either consistent (batch 8032411_MHD 30.6.2021), occasional (batch 9034511_MHD 11.2022), or no (batch 1032821_MHD 31.07.2024) *vapA* induction. Manufacturer information retrieved via the vendor (Sarstedt) stated that they were not aware of any alterations in the production process between these batches. This indicated that apparently subtle changes in the product process may have strong effects on the interaction of the product with microorganisms.

In an effort to make sure that VapA induction was not an isolated feature of just one virulent *R. equi* strain (strain ATCC 33701, isolated in Canada), additional well-established *R. equi* strains isolated from pneumonic foals at different places and times, strains 103 (Canada) and 85F (Japan), were tested using a reliably inducing tube batch. These isolates also showed the induction phenomenon (Fig. 1D). Strain 33701, previously used to study *vapA* expression regulation (27, 28), was used in further experiments unless indicated otherwise.

### The inducing plastic factor is unlikely to be a leachable or an extractable compound.

**FIG 2:**
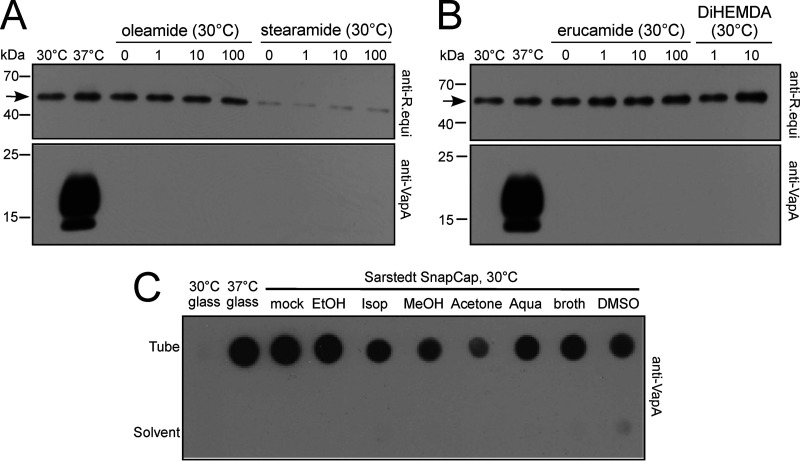
VapA expression at 30°C is not induced by supplements commonly used in plastic manufacturing. (A and B) A 30°C overnight culture of *R. equi* 33701 was diluted into fresh BHI at an OD_600_ of 0.1 and grown for 18 h at 30°C or 37°C in glass tubes supplemented with (A) oleamide or stearamide or (B) erucamide or di-HEMDA at the indicated concentrations (micromolar units). Lanes labeled “0” contain the respective carrier controls. Sample buffer extracts of equal numbers of bacteria were analyzed as for Fig. 1. Top panels show an approximately 6-kDa loading control protein detected by *R. equi* antiserum. The protein was less strongly expressed in the presence of ethanol. (C) An overnight culture of *R. equi* 33701 at 30°C was diluted into fresh BHI broth at an OD_600_ of 0.1 and grown for 18 h in Sarstedt Snap Cap tubes that had been pretreated for 2 days at 21°C as indicated or in untreated glass tubes. Bacteria were grown at 30°C for 18 h (as for Fig. 1), and the bacteria were analyzed (Tube) or shaken in glass tubes supplemented with 5% (by volume) of the respective 2 day wash supernatant solution (Solvent). EtOH, ethanol; Isop, isopropanol; MeOH, methanol; DMSO, dimethyl sulfoxide. The immunoblots are representative of three independent experiments.

To identify the expression-inducing element, we chose to analyze plastic surface-associated slip agents which had previously been implicated in interference with different biological processes. Slip agents are often nature-identical substances that are added during plastic manufacture to reduce the frictional resistance of product surfaces. They form a lubricant layer at the polymer-liquid interface, and therefore, they were prime candidates for diffusible agonists (17). The following frequently used slip agents were tested. (i) The first is oleamide, which is also a signaling molecule in cerebrospinal fluid (29). At 100 μM, it inhibited hMAO-A and -B *in vitro* by 80 to 90% (13). Jug et al. (29) showed that oleamide is a major leachable from labware plastic. Here, oleamide did not induce *vapA* expression even at 100 μM (Fig. 2A). (ii) Similarly, a 100 μM concentration of the slip agent stearamide, which is produced by mushrooms and which also has antimicrobial activity, did not induce *vapA* expression (30) (Fig. 2A). (iii) Erucamide is routinely used in plastic manufacturing (13) and can also act as an angiogenic factor (31). Here, at 100 μM, it did not stimulate VapA induction (Fig. 2B). (iv) The slip agent di-HEMDA [di(2-hydroxyethyl)methyldodecylammonium] as used in the Holt lab study (13) also did not stimulate VapA induction. Addition of up to 10 μM did not promote VapA induction. For comparison, human hMAO-B was inhibited by 80% in 10 μM di-HEMDA, and concentrations higher than 10 μM inhibited *R. equi* growth. In summary, none of these prime candidate compounds induced *vapA* expression.

To further test whether substances leaching from plastic would induce *vapA* expression, we washed a presumably present leaching compound from plastic tubes and transferred it to glass tubes, as described in reference 13. In detail, sterile broth media which had been shaken overnight at 30°C in plastic tubes were used for subsequent bacterial culture in glass tubes. These conditioned media did not induce *vapA* expression at 30°C during the next 24 h (data not shown). To further force the release of potentially relevant leachates or extractables, we preincubated unused plastic tubes for 2 days with water or sterile broth or any of the organic solvents ethanol, methanol, isopropanol, acetone, and dimethyl sulfoxide (DMSO). Previous studies recommended this treatment for efficient release and collection of transferable, more hydrophobic compounds (13, 29). However, these pretreatments did not reliably reduce the ability of plastic to induce VapA expression in subsequent *R. equi* cultivation at 30°C; i.e., the reduced expression seen in this experiment after acetone pretreatment (Fig. 2C) was not reproduced in other experiments. In a converse approach, the organic eluates were added to fresh brain heart infusion (BHI) broth at 5% (by volume) in glass tubes for growth experiments at 30°C, which never led to any *vapA* induction (Fig. 2C). Therefore, extraction of a putative inducer did not reduce the expression effect, nor was a putative inducer transferred into fresh (glass) tubes.

In summary, induction could occur with different types of tubes representing entirely different compositions and modes of production (polypropylene, polystyrene, polyethylene terephthalate) and was not influenced by prewashing the tubes with organic compounds. No inducing leachate could be transferred from one tube to another, and purified slip agents that had previously been identified to interfere with biological reactions did not induce *vapA* expression either. These data indicated that leachates are not the sought-after inducers but that some nonmobilizable property of the system induced *vapA* expression.

### Induction ability of Sarstedt polypropylene is lost upon heating.

**FIG 3:**
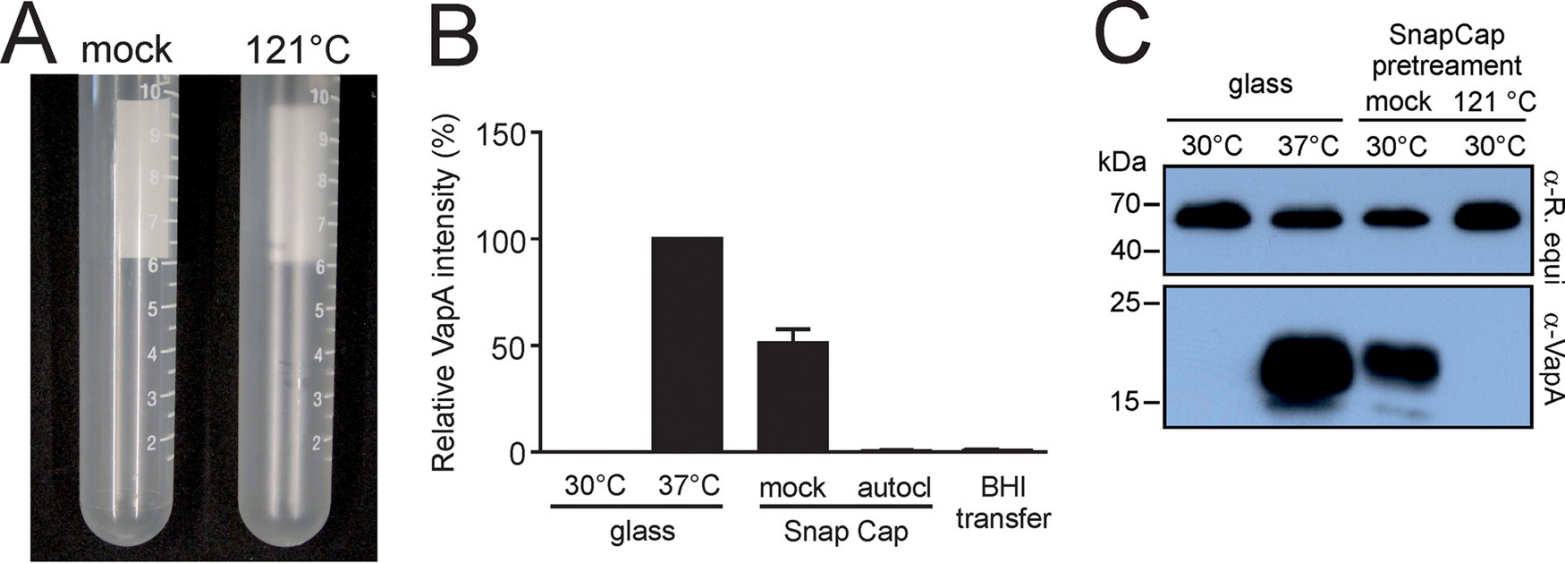
Sarstedt Snap Cap culture tubes lose the ability to induce VapA expression during heating at 121°C. (A) Sarstedt Snap Cap tubes left for 2 h at ambient temperature (mock) or 121°C dry heat are shown. Note the increased turbidity of the heated tube. (B) Autoclaving. An overnight culture of *R. equi* 33701 at 30°C was diluted into fresh BHI broth at an OD_600_ of 0.1 and grown for 18 h in glass tubes or Snap Cap tubes that had been untreated (mock) or autoclaved (autocl) at 121°C. “BHI transfer” represents a sample in which the BHI broth from an autoclaved Snap Cap tube was transferred into a glass tube and *vapA* expression during growth at 30°C was assessed. In all cases, *vapA* expression was quantified by VapA Western blotting and blot scanning, and the blot signal for the glass/37°C sample was set as 100% in each experiment. Three independent experiments were performed, and the means and standard deviations (SD) are shown. (C) Dry heat. A Western blot representative of three experiments developed with anti-VapA shows samples as generated for panel B but in regular glass tubes or in Snap Cap tubes pretreated (121°C) or not (mock) in a dry heat cabinet for 120 min. Growth temperature was 30°C or 37°C, as indicated. As a loading control, we used rabbit antiserum to *R. equi* as for Fig. 2A and B.

Hypothesizing that the structure of plastic rather than its compounds had an effect on *R. equi* gene expression, we heated the tubes, altering the structure of polypropylene to a more crystalline state and causing an increase in haze and stickiness (32) (Fig. 3A). Heating of Sarstedt Snap Cap tubes in dry heat at 121°C for 120 min (Fig. 3B) or in an autoclave (Fig. 3C) led to a complete loss of their capability to stimulate VapA production, and transferring BHI broth that had been autoclaved within the PP tube to a glass tube to serve as fresh growth medium for *R. equi* did not result in *vapA* induction (Fig. 3A). These data are best interpreted as showing that the inducing factor is not some leachable substance from the plastic ware but a physical tube property. They also agree well with the observed loss of induction after altering the tube surface by siliconization (Fig. 1C) and with the concurrent inability to remove or transfer an active component by incubation of tubes with solvents (Fig. 2C).

### Stimulation of VapA expression by plastic is regulated through the VirR/VirS pathway.

**FIG 4:**
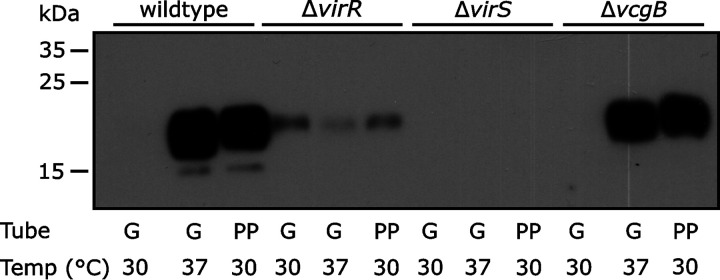
Induction of VapA expression at 30°C depends on the *virR*/*virS* regulatory system. Overnight cultures of *R. equi* 103 and its isogenic deletion mutants 103/Δ*virR*, 103/Δ*virS*, and 103/Δ*vcgB*, grown at 30°C, were diluted into fresh BHI broth at an OD_600_ of 0.1 and grown for 18 h in glass tubes (G) or Sarstedt Snap Cap tubes (PP) at the indicated temperatures. Extracts of equal numbers of bacteria were analyzed for *vapA* expression by Western blotting. A representative blot from three independent experiments is shown.

Even though the precise signaling mode for *vapA* induction was unclear, we wondered whether induction was dependent on the proteins which regulate *vapA* transcription in the physiological setting. Normally, *vapA* expression is increased by raising the growth temperature to about 37°C and, to some degree, by lowering the pH (10, 11, 28, 33). It is noteworthy that the culture medium pH did not decrease in any tube throughout the experiments. Temperature and pH are relayed in an unknown fashion to the LysR-type transcription factor VirR, whose gene is also localized to the virulence plasmid (9, 10, 27, 28). VirR is constitutively expressed, most likely through the principal sigma factor (26), and cooperates with the orphan two-component response regulator VirS in *vapA* regulation (34) (Fig. 4). VirS expression is induced when VirR is activated directly or indirectly by signals such as high environmental temperature (9). VirS then promotes transcription of *vapA* (Fig. 4). Activation of VirS may uncanonically occur without phosphorylation by a histidine kinase (10). In summary, the transcription of *virR* is largely temperature independent, whereas expression of *virS* is dependent on high temperature.

**FIG 5:**
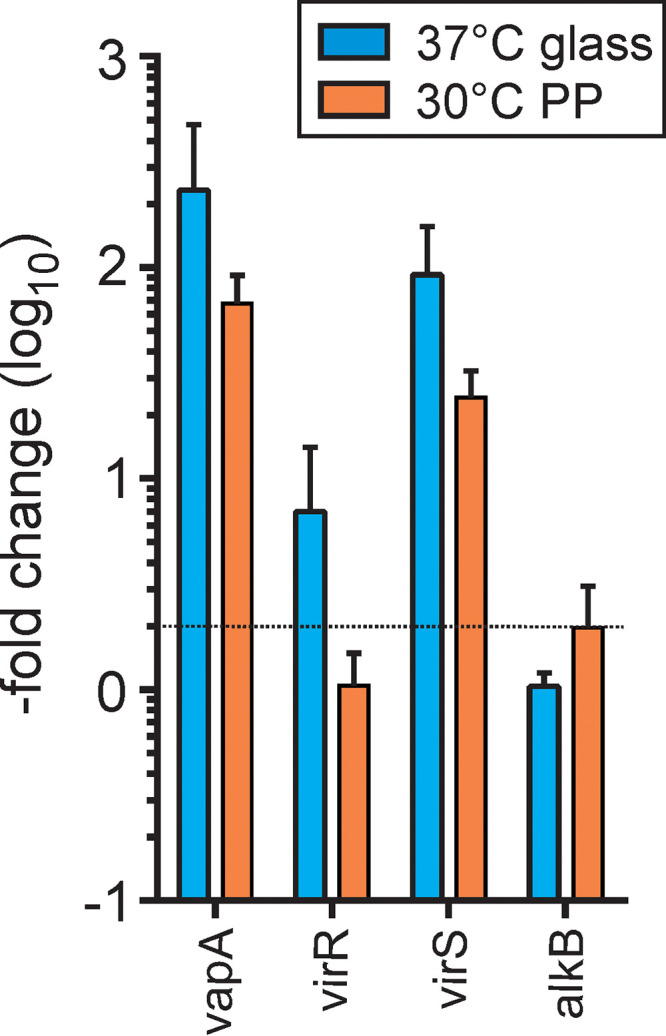
Quantitative real-time PCR of genes involved in *vapA* expression. Total RNA of *R. equi* 33701 was isolated from cultures grown for 18 h in glass tubes at 30°C or 37°C, as indicated, or in Sarstedt Snap Cap polypropylene (PP) tubes at 30°C and further analyzed by quantitative real-time PCR. Values shown indicate the fold change in *vapA* mRNA abundance (log_10_) between bacteria from 30°C/glass cultures and from the respective test sample. *alkB* denotes the transcripts from an alkane monooxygenase gene. The dotted line marks the “no change” horizon. Data are means and standard deviations from three independent experiments.

We used *virR* or *virS* deletion mutants of the virulent strain 103 (35) and tested if plastic-driven *vapA* induction occurred in the absence of either gene, i.e., whether there is an alternative route of expression stimulation. *vapA* expression was strongly induced in wild-type bacteria grown at 37°C in glass tubes, and expression was strongly reduced in a Δ*virR* and virtually absent in a Δ*virS* background (Fig. 5). The very weak *vapA* expression in the Δ*virR* background was similar at 30°C and 37°C, hinting once more that VirR is the constitutively expressed integrator of environmental signals. A *vcgB* (*vapA*-coexpressed gene B) deletion mutant (36), constructed in strain 103 using the same strategy as for the Δ*virR* and Δ*virS* strains (35), was used as a control strain for possible genetic manipulation side effects and behaved like wild-type bacteria (Fig. 5). Clearly, the expression patterns of plastic-grown *R. equi* were as strongly influenced by the mutations as expression at 37°C in glass-grown cells. In summary, plastic-mediated *vapA* induction at 30°C followed, at least in central parts, the same pathway as high-temperature induction in glass.

**FIG 6:**
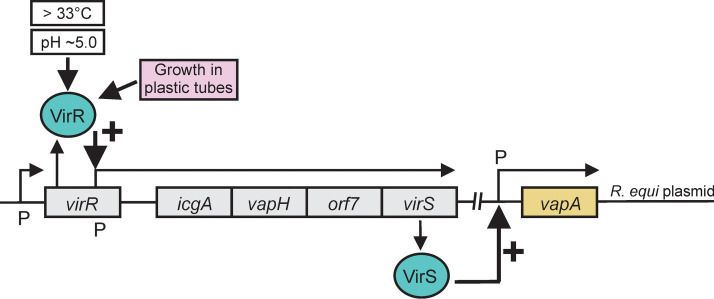
Working model of VapA transcriptional regulation modified from reference 10. Increased temperature and moderately low pH act on the constitutively expressed LysR-type transcription regulator VirR in an unknown way. VirR then stimulates transcription of the *icgA* operon, including the *virS* gene. The newly produced VirS protein, an orphan two-component signal transduction response regulator, and then strongly upregulates *vapA* transcription. We show in this study that plastic-mediated induction of *vapA* expression at 30°C uses at least part of the same pathway through VirR, although the high-temperature requirement is circumvented. P, promoter.

To analyze gene regulation with a technology independent of immunoblotting, we quantified *vapA* transcription during cultivation at 30°C or 37°C in glass or in Sarstedt Snap Cap tubes using quantitative PCR (qPCR). *vapA* transcription in *R. equi* grown in glass at 37°C was induced 233-fold compared to expression at 30°C, similar to reports by others (36). The transcription increase of *virR* at 37°C in glass was a more moderate 7-fold, and that of *virS* expression was 93-fold, all in good agreement with previous observations (26). The chromosomal *alkB* gene, coding for an alkane monooxygenase, was included as a negative control (Fig. 6).

In comparison, during growth in reliably “bioactive” Sarstedt polypropylene tubes, there was no increase in the *virR* mRNA level, whereas the *virS* mRNA level increased 25-fold and *vapA* mRNA increased a substantial 68-fold (Fig. 6). Therefore, the *vapA* transcription patterns during growth in glass at 37°C paralleled those of growth in plastic at 30°C, even though the induction levels upon plastic contact were lower than those in glass. In summary, this indicated that the same regulatory proteins participate in regulation of *vapA* expression during growth in plastic at 30°C as at elevated temperatures in glass (Fig. 4).

### Conclusion.

We present here the first evidence that contact with human-made plastic can dramatically change the regulation of a central virulence gene in a pathogenic bacterium. Occurrence of potentially pathogenic bacteria on environmental plastic has been described (20–23), and the possibility of plastic-driven pathogen gene regulation has been discussed (19, 37) but remained speculative. Although we did not identify a hypothetical “plastic sensor” in this study, our data show that expression induction at 30°C occurred through transcriptional regulation by the virulence plasmid-encoded VirR and VirS proteins, which also participate in the high-temperature-and-low-pH mode of expression induction (10). We have further shown that induction was not attributable to any of several plastic slip agents which can interfere with biological reactions. We present evidence that there was no leakage of a bioactive plastic substance from within plastic into the growth medium and neither was a potential soluble expression-stimulating factor extracted with either of several organic solvents. Heating of the tubes destroyed their capability to induce *vapA* expression and also caused an increase in surface polarity (38), microscopic changes in surface structure, and changes in crystallinity (39), yet the precise cause of loss of *vapA* induction remains enigmatic. However, together with the Teflon and silicone tube experiments, our data point to a role of tube surface characteristics in *vapA* induction.

It is not clear whether the induction phenomenon described here bears any relevance to *R. equi*-caused disease, yet it is interesting that *R. equi* growing on plastic catheters can cause bacteremia in cancer patients (40). Additionally, reports by others show that rhodococci, as ubiquitous environmental degraders, can be closely associated with environmental plastic (41–43). Collectively, our data allow at least four relevant conclusions: (i) contact with plastic can strongly influence the expression of bacterial virulence genes, (ii) several kinds of plastic can regulate expression of the same gene, (iii) induction is dependent on surface characteristics of plastic, and (iv) phenomena like this could further increase the danger when plastic microparticles with “artificially” induced virulence or metabolic genes are ingested. Taken together with the recent observations that ingested microplastics can change the integrity of intestines and the composition of intestine-associated microbiotas and increase the carriers’ inflammatory status (44, 45), our results show that all these factors can cooperatively compromise the health of wildlife.

## MATERIALS AND METHODS

### Chemicals and antibodies.

All standard chemicals were of analysis grade and purchased form Sigma-Aldrich, Carl Roth, or Fisher Scientific. Oleamide (Sigma-Aldrich, catalog no. O2136) was dissolved at 100 mM in DMSO, erucamide (Sigma-Aldrich; catalog no. 90082) at 50 mM in isopropyl alcohol, and stearamide (Cayman Chemical, catalog no. 21087) at 50 mM in ethanol. di(2-hydroxyethyl)methyldodecylammonium (di-HEMDA) was purchased from Watson International, Ltd. (Kunshan City, Jiangsu, China), and dissolved at 100 mM in Milli-Q water. Peroxidase-coupled goat anti-mouse secondary antibody (catalog no. 115-035-062, batch 134308) was from Jackson ImmunoResearch. The manufacturers of plastic tubes, catalog numbers, and batch numbers are listed in Table 1.

**TABLE 1:** Tubes used for cultivation of *R. equi*

Type	Material	Manufacturer	Catalog no.	Batch no.
Tube,^*a*^ 13 mL	PP	Sarstedt	62.515.028	8032411
Tube, 15 mL	PP	Sarstedt	62.554.502	0040821
Tube, 13 mL	PTFE^*b*^	Fisher Scientific	16255971	
Centrifuge tube, 11 mL	PC	Nunc	347708	167259
Centrifuge tube, 15 mL	PET	Corning	430053	09118003
Conical tube, 15 mL	PP	Eppendorf	0030.122.151	H176351H
Conical tube, 15 mL	PP	Falcon	3552096	15418105
Centrifuge tube, 15 mL	PP	VWR	5250604	180606058
Tube, 14 mL	PP	Greiner Bio One	187261	E1511359
Tube, 15 mL	PP	Greiner Bio One	188271	E161239J
Centrifuge tube, 15 mL	PS	TPP	91115	20120447
Glass tubes, 16 by 160 mm	Glass	DKW Life-Sciences	26131210	
Glass tube lids	Aluminum	Schütt Labortechnik	3.620.713	

a Referred to as “Snap Cap.”

b Polytetrafluoroethylene (Teflon).

### Bacteria and cultivation.

*R. equi* strains 103, 103/Δ*virR*, 103/Δ*virS,* and 103/Δ*vcgB* (35) were kindly provided by John F. Prescott (University of Guelph, ON, Canada) and *R. equi* 85F (46) by Axel Hartke (University of Caen Normandy, Caen, France). *R. equi* ATCC 33701 was from the American Type Culture Collection (Manassas, VA, USA). Bacteria were maintained on BBL brain heart infusion (BHI; Becton Dickinson, catalog no. 90000-060) agar plates at 30°C. For experiments, bacteria were grown overnight in glass tubes in a 10-mL BHI starter culture under noninducing conditions (pH 7, 30°C). This pH did not decrease (and, therefore, possibly induce *vapA* expression) throughout the cultivation. The starter culture was diluted into 3 mL of fresh BHI at an optical density at 600 nm (OD_600_) of 0.1 and grown further at 30°C or 37°C for 18 h at 200 rpm in glass or plastic tubes, as indicated. Polytetrafluoroethylene tubes did not come with a serial cap and were covered with sterile aluminum foil during cultivation. Where indicated, glass tubes and Snap Cap tubes were covered with a silicone layer by using 5 mL organopolysiloxane in heptane (Sigmacote; Sigma-Aldrich-Merck, catalog no. SL2) to fill the tubes, and the tube was swirled to cover the whole inner surface. The formation of a silicone layer was almost instantaneous. Tubes were dried overnight to evaporate the heptane solvent. Before use, tubes were rinsed repeatedly with Milli-Q water. Where indicated, Sarstedt 13-mL tubes were autoclaved with aluminum foil instead of their plastic caps because these were heat sensitive. The sterile caps were placed back on the tubes after autoclaving.

### Analysis of VapA expression.

Bacterial suspensions, cultivated as described above, were carefully removed from the culture tubes, transferred into fresh tubes, harvested by centrifugation for 3 min at 7,000 × *g* in a microcentrifuge, washed once with phosphate-buffered saline (PBS), and resuspended in PBS. Bacteria equivalent to OD_600_ of 0.1 (1.5 × 10^7^ bacteria) for Western blotting or 0.01 (1.5 × 10^6^ bacteria) for dot blot analysis were heated in SDS sample buffer for 10 min at 95°C and centrifuged for 3 min at 7,000 × *g*, and the supernatants were separated by SDS-gel electrophoresis and transferred onto nitrocellulose membranes (immunoblotting; Carl Roth [Karlsruhe, Germany], catalog no. HP40.1) or directly spotted onto nitrocellulose membrane in equal volumes (immuno-dot blotting). Membranes were incubated with murine anti-VapA primary monoclonal antibody (Santa Cruz Biotechnology; clone E-6, sc-390576, batch B0216) at a dilution of 1:1,000 for 1 h at ambient temperature (dot blotting) or 18 h at 4°C (Western blotting) and developed with horseradish peroxidase (HRP)-coupled secondary antibody (Dianova, catalog no. 115-035-062) at 1:10,000 for 1 h at ambient temperature followed by enhanced chemiluminescence (SuperSignal West Pico Plus; Thermo Scientific) and exposure to X-ray film (Super RX-N; Fujifilm, Japan). This VapA monoclonal antibody recognizes exclusively VapA, as validated by the complete absence of signal in *vapA* deletion strain samples (6). Where indicated, blots were probed with a custom-made polyclonal mouse antibody raised against formaldehyde-fixed *R. equi* 103- (strain isogenic to strain 103 but cured of the virulence plasmid). For solvent treatment of plastic tubes before cultivation of bacteria, 6 mL of the indicated solvents was used to fill the closed tubes, and the tubes were shaken at 21°C for 48 h in a rotary shaker at 60 rpm. Solvents were collected in glass tubes, and the treated tubes were washed three times with distilled Milli-Q water before 3 mL fresh BHI broth was added and inoculated with bacteria as indicated. In parallel, glass tubes were filled with 3 mL BHI broth plus 150 μL of one of the “solvent eluates” and used for inoculation with bacteria to determine whether the eluates would have any effect on VapA induction. At this concentration of solvents, growth of the bacteria was not affected.

### RNA isolation and cDNA synthesis.

*R. equi* total RNA was isolated from cultures grown for 18 h in glass tubes at 200 rpm and 30°C or 37°C, as indicated, or from cultures grown in Sarstedt Snap Cap tubes at 30°C. Of this, 1 × 10^9^ cells were harvested by centrifugation for 5 min at 17,000 × *g*, resuspended in 1 mL of RNAlater solution (Thermo Scientific, catalog no. AM2070) to stabilize RNA, and stored at 4°C. Total RNA was isolated using a RiboPure-Bacteria kit (Invitrogen, catalog no. AM1925) according to the manufacturer’s instructions, except for the disruption step, which was performed in a precooled TissueLyser LT instrument (Qiagen, catalog no. 85600) for 20 min at 50 Hz. To eliminate DNA, the DNase I treatment step was performed. RNA yield and purity were determined by UV absorbance in a spectrophotometer at 260 nm and 280 nm. An *A*_260_/*A*_280_ ratio between 1.8 and 2.0 was considered indicative of pure RNA. cDNA was synthesized from 1 μg of total RNA using the iScript cDNA synthesis kit (Bio-Rad, catalog no. 1708891) according to the manufacturer’s instructions in a Mastercycler personal thermal cycler (Eppendorf) applying the following Bio-Rad standard protocol: priming for 5 min at 25°C, reverse transcription for 20 min at 46°C, reverse transcriptase inactivation for 1 min at 95°C, and a hold at 4°C.

### Real-time quantitative PCR (RT-qPCR).

Before the gene expression assays, primer pairs were validated by preparing a 5-point standard curve (10-fold dilution series) of the positive control (37°C glass). Reaction efficiency (E) was calculated for each pair as 10^1−^*^s^*, where *s* is the slope of the standard curve. Assay specificity was verified by running a melt curve (47). The oligonucleotides and the E values are listed in Table 2. The quantitative real-time PCR was performed using SsoFast EvaGreen Supermix (Bio-Rad, catalog no. 172-5200) in a 10-μL reaction volume. The cycling conditions for amplification in a Bio-Rad CFX96 real-time PCR system controlled by the CFX 3.1 software are listed in Table 3. *gapdh* and *gyrB* were used as reference genes for normalization. Relative gene expression was calculated as described in reference 48.

**TABLE 2:** Oligonucleotides used in this study and their reaction efficiencies

Oligonucleotide	Sequence (5′-3′)	Efficiency (%)^*a*^
alkB-FW	AAGTGGCCGTAGAACGACTG	105.1
alkB-RV	AACTCGGCCACAAGAAGGAC	
GAPDH-FW	TCACCGTCAACCTGTCGAAG	107.6
GAPDH-RV	ACTTCAGGATGCCCTTCAGC	
gyrB-FW	GATCTACATCGTGGAGGGCG	99.2
gyrB-RV	GACGTTGATGATCTTGCCGC	
icgA-FW	GATCTAGGTCTCAAGCCCGC	98.1
icgA-RV	CAAAAACCCAGGGCAAGACG	
orf3-FW	ACCAAGCCAGCCTTATCGTC	105.5
orf3-RV	GAGGATGGGGTGTTCTTCGG	
sig70-FW	CCACCAACCTCTTCCTGGAC	107.7
sig70-RV	GTCGTGGTAGATCTGCTCGG	
vapA-FW	AGAGCAGCAGTACGACGTTC	109.3
vapA-RV	TCGCCATCGAAGACCTTTCC	
vapH-FW	ACACACGACCTGCCTACTTG	95.3
vapH-RV	TGTCACTGTGAGTCGATGGC	
virR-FW	GAGACCCACCAAAGAGACCG	105.8
virR-RV	CGGTTTGCTAGAAACGCGAG	
virS-FW	TCGACCTGCTCGCATACTTC	108.4
virS-RV	TGACGTGTACAGTGACCGTG	

a Reaction efficiency was determined as stated in Materials and Methods.

**TABLE 3:** Cycling conditions for real-time PCR

Cycling step	Temp (°C)	Time (s)	No. of cycles
Enzyme activation	95	30	1
Denaturation	95	5	40
Annealing/extension	60	5	40
Melt curve	65–95^*a*^	5 (per step)	1

a In 0.5°C increments.
